# Imaging video plethysmography shows reduced signal amplitude in glaucoma patients in the area of the microvascular tissue of the optic nerve head

**DOI:** 10.1007/s00417-020-04934-y

**Published:** 2020-09-22

**Authors:** Ralf-Peter Tornow, Radim Kolar, Jan Odstrcilik, Ivana Labounkova, Folkert Horn

**Affiliations:** 1 Department of Ophthalmology, Friedrich-Alexander-University of Erlangen-Nuremberg, 91054 Erlangen, Germany; 2 Department of Biomedical Engineering, Faculty of Electrical Engineering and Communication, Brno University of Technology, 616 00 Brno, Czech Republic

**Keywords:** Retinal plethysmography, Glaucoma, Perfusion, Blood volume, Blood flow

## Abstract

**Purpose:**

To measure parameters of the cardiac cycle-induced pulsatile light absorption signal (plethysmography signal) of the optic nerve head (ONH) and to compare parameters between normal subjects and patients with different stages of glaucoma.

**Patients and methods:**

A recently developed video ophthalmoscope was used to acquire short video sequences (10 s) of the ONH. After image registration and trend correction, the pulsatile changing light absorption at the ONH tissue (excluding large vessels) was calculated. The changing light absorption depends on the pulsatile changing blood volume. Various parameters, including peak amplitude, steepness, time-to-peak, full width at half maximum (FWHM), and pulse duration, were calculated for averaged individual pulses (heartbeats) of the plethysmography signal. This method was applied to 19 healthy control subjects and 91 subjects with ocular hypertension, as well as different stages of primary open-angle glaucoma (17 subjects with ocular hypertension, 24 with preperimetric glaucoma, and 50 with perimetric glaucoma).

**Results:**

Compared to the normal subjects, significant reductions (p < 0.001) in peak amplitude and steepness were observed in the group of perimetric glaucoma patients, but no significant difference was found for time-to-peak, FWHM, and pulse duration. Peak amplitude and steepness showed high correlations with RNFL thickness (p < 0.001).

**Conclusions:**

The presented low-cost video-ophthalmoscope permits measurement of the plethysmographic signal of the ONH tissue and calculation of different blood flow-related parameters. The reduced values of the amplitude and steepness parameters in perimetric glaucoma patients suggest decreased ONH perfusion and blood volume. This outcome is in agreement with results from other studies using OCT angiography and laser speckle flowgraphy, which confirm reduced capillary density in these patients.

Registration site: www.clinicaltrials.gov, Trial registration number: NCT00494923

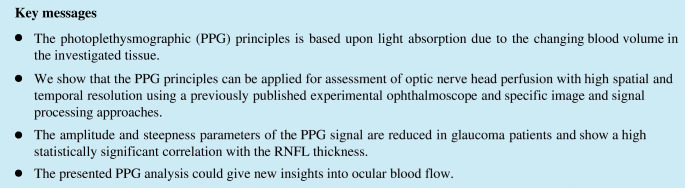

## Introduction

Knowledge of the perfusion of the optic nerve head (ONH) is important not only in the diagnosis of ocular diseases such as glaucoma and diabetic retinopathy but also in basic research to better understand the aetiology of eye diseases. There is a huge variety of different methods to assess ocular blood flow (OBF) [1]. Techniques differ in the underlying basic principles, fields of view (e.g. macular region, ONH, single vessels), measured location (large vessels or capillaries), and assessed parameters.

Optical methods to assess ocular blood flow are based on two basic principles: light absorption by blood components or light scattering by moving blood components.

In early measurements of retinal blood flow, non-imaging light absorption–based methods were used [2–6]. With light absorption–based methods, only the changing fraction of the blood flow can be measured. This changing fraction can occur due to provocation (increased intraocular pressure (IOP), flickering light, changing intensity after injection of dyes) and also due to the cardiac cycle–induced pulsatile changing blood volume in the investigated region of interest (ROI). With the development of highly light-sensitive imaging detectors, non-invasive imaging methods to measure cardiac cycle–induced changing blood volume could also be realized [7–11]. Examples of imaging dye injection–based methods are fluorescein and indocyanine green angiographies, two important techniques for clinical applications to visualize and assess ocular blood flow. However, due to the dye injection, these methods are invasive and their popularity is decreasing due to the development of new, advanced, dye-free imaging techniques. So far, non-invasive light absorption–based blood flow measurement methods have no clinical importance.

There are also methods that measure the movement of blood cells using coherent light and interference techniques such as the Doppler effect or speckle methods. Doppler techniques and speckle methods represent two ways of looking at the same physical phenomenon [1, 12, 13]. Scanning laser flowmetry (SLF), the combination of a laser Doppler flowmeter with a scanning laser system, allows visualization of the perfusion of the retina and ONH [14]. However, SLF has only slight clinical importance. Two non-invasive optical methods with a widespread clinical application are OCT angiography (OCT-A) and laser speckle flowgraphy (LSF). Both methods can be realized as imaging methods and are available as commercial instruments.

Optical coherence tomography angiography (OCT-A) is a functional extension of OCT that provides information/visualization of perfused retinal (and choroidal) vasculature without the need for dye injection [15]. Blood flow–related parameters are calculated from several consecutive B-scans acquired at the same location. There are different algorithms to calculate parameters from raw data [16–20]. The most important parameters for clinical applications are capillary density and blood flow index [21]. Recent studies show that the OCT-A technique is able to clearly visualize perfusion changes in the microvascular network inside ONH and in the peripapillary area [16, 22–25]. However, the waveform of the pulsatile component of the blood flow cannot be assessed by OCT-A due to the relatively long acquisition time (several seconds).

Laser speckle flowgraphy (LSF) can also be applied to measure perfusion-related parameters. The method is based on changed speckle statistics for moving targets, which can be evaluated by computing the image contrast. The primary outcome is the variation in time of the mean blur rate (MBR), which is a dimensionless parameter depending on the erythrocyte velocity [26]. MBR shows a pulsatile behaviour that reflects the pulsatile blood flow. From the waveform of MBR, additional parameters can be calculated—average MBR (MBR_avg_), the flow index, and the flow acceleration index [27, 28]. LSF shows a reduction in the microcirculation in the ONH in glaucoma patients [29, 30]. An advantage of LSF, compared with OCT-A, is the possibility to measure the waveform of the pulsatile signal, which can be changed in diseased eyes. This was shown for patients with glaucoma [28, 31].

In this paper, we describe the application of a light absorption–based method to measure the cardiac cycle–induced pulsatile changing blood volume in the ONH using a recently developed video ophthalmoscope. The main outcome is the photoplethysmographic signal of the tissue of the ONH and derived parameters, which are possibly useful as new biomarkers.

The method described here has some advantages compared with established methods. Due to the development of very light-sensitive image detectors (CCD and CMOS cameras) and compact and effective light sources (LED), such an instrument can be realized relatively simply and cost-effectively using mainly commercial components available from stock. Additionally, it can be made battery (or USB)-powered and portable [32, 33]. The measurement (acquisition of a video sequence) is fast and non-invasive. Once the video sequence is acquired and stored, the plethysmographic signal can be calculated offline with high temporal and spatial resolutions at any position and with any size within the acquired field of view. Therefore, it is possible to assess signals from different positions along the blood vessel system consisting of arteries, arterioles, capillaries, venules, veins in the ONH, and peripapillary area from a single measurement. An example of the comparison of signals from the entire ONH and a vein is given in [32].

Here, we concentrate on the plethysmographic signal of the capillaries of the ONH. The proposed method was applied to 110 subjects (19 normal subjects and 91 glaucoma patients) to compare parameters of the plethysmographic signal of the ONH between glaucoma groups and with the retinal nerve fibre layer (RNFL) thickness assessed by optical coherent tomography (OCT).

## Methods

### Procedures/subjects

The study included 19 healthy subjects and 91 patients of the Erlangen Glaucoma Registry (www.clinicaltrials.gov, NCT00494923). All patients and healthy subjects had long experience with ophthalmological examinations. The Erlangen Glaucoma Registry is a clinical registry for cross-sectional and longitudinal observation of patients with open-angle glaucoma (OAG) or glaucoma suspect and was founded in 1991. The primary aim is the evaluation of the diagnostic and prognostic validity of morphometrical, sensory, and hemodynamic diagnostic procedures. The inclusion/exclusion criteria, the definition of the gold standard, the schedule, and the type of examinations are defined in a protocol that was approved by the local ethics committee. The study followed the tenets of the Declaration of Helsinki for research involving human subjects and informed consent, and agreements for data collection were obtained from all participants of the study. All control subjects and patients were thoroughly examined by slit-lamp inspection, applanation tonometry, fundoscopy, gonioscopy, standard automated perimetry (SAP), and papillometry. Optic disc evaluations of patients and controls were based on 15-degree colour images (Zeiss tele-centric fundus camera, Carl Zeiss Meditec). Glaucomatous damage was defined as a glaucomatous appearance of the optic nerve head, e.g. an unusually small neuroretinal rim and notching, or cup/disc ratios being higher vertically than horizontally. Optic disc evaluations were independently performed by 2 glaucoma experts of the EGR. A third clinical glaucoma expert was consulted to decide in case of discrepancy [34]. In addition, a 24-h intraocular pressure curve (6 determinations) was measured in all patients. Table 1 includes the IOP at the time of video acquisition and the mean of the daily profile. The mean value of IOP did not differ between groups, with the exception of the OHT (ocular hypertension group) group, which showed slightly larger values. For some of the measurements, pupils were dilated using Mydriatikum Stulln (Pharma Stulln GmbH, Germany) and Neosynephrin (URSAPHARM Arzneimittel GmbH, Germany). Criteria for glaucoma diagnosis were an open anterior chamber angle and glaucomatous appearance of the ONH, including an unusually small neuroretinal rim area in relation to the ONH size and cup-to-disc ratios being higher vertically compared with horizontal measurements [35]. All subjects of the EGR had annual examinations. During an 8-month period, all attending subjects additionally had an examination with a video ophthalmoscope.

**Table 1:** Subject data. Patient groups: *OHT*, ocular hypertension; *prep*, preperimetric glaucoma; *perim*, perimetric glaucoma; *norm*, normal subjects

Patient group	Number of subjects	Male/female	Age (years)	Refraction (dpt)	Visus	RNFL mean thickness (μm)	Pulse rate (1/min)	IOP (at time of examination)	IOP (average of 24-h profile)
Norm	19	9/10	65.03 ± 12.84	− 0.38 ± 2.69	0.93 ± 0.14	92.45 ± 9.81	59 ± 10	15.1 ± 2.7	13.9 ± 1.9
OHT	17	8/9	63.06 ± 10.45	0.32 ± 1.78	0.86 ± 0.18	92.82 ± 9.62	65 ± 11	17.3 ± 2.6	15.9 ± 2.9
Prep	24	11/13	67.91 ± 8.58	− 0.66 ± 1.96	0.87 ± 0.13	76.10 ± 14.32	56 ± 7	14.3 ± 3.2	14.1 ± 2.9
Perim	50	29/21	67.97 ± 10.64	− 1.02 ± 2.73	0.75 ± 0.21	61.36 ± 11.54	62 ± 11	13.2 ± 4.1	12.7 ± 3.5

The pulse rate presented here is calculated from the video sequence (see below)

The means of the best-corrected visual acuity (VA) in the 4 groups of the study are given in Table 1. The minimum VAs were 0.7 in the normal and OHT groups, and 0.63 in the preperimetric glaucoma group. In the perimetric glaucoma group, there were 6 subjects (16%) showing VAs less than 0.6 due to glaucoma. We included all subjects that were able to control fixation; refraction was compensated by the instrument.

All eyes included in the study had clear optic media. On the day of the examination, the intraocular pressure was equal to or less than 22 mmHg. Exclusion criteria were all eye diseases other than glaucoma, diabetes mellitus, colour vision anomalies, and a myopic refractive error exceeding − 12 dioptres. The age was 40 years and older. The age and the pulse rate (calculated from pulse length, see below) did not differ between study groups (Kruskal-Wallis test).

### Perimetry

All subjects underwent visual field testing with standard white-on-white perimetry using a computerized static projection perimeter (Octopus-G1, Interzeag, Switzerland). All patients had the 3-phase protocol (full threshold) to calculate the ‘corrected loss variance’ (CLV). Those subjects with rates of false-positive or false-negative responses higher than 12% were not included in this study. Similar to what has been suggested earlier [36], a white-on-white perimetry was classified as a ‘non-normal’ visual field when one of the following was present: (a) at least three adjacent test points in the superior or inferior hemifield having a probability of ≤ 5% and with one test point with a defect of ≤ 1%, or (b) at least two adjacent test points having a probability of ≤ 1%. These criteria had to be confirmed in at least the two most recent Octopus measurements at the same test location. SAP was used to separate the glaucoma cohort into perimetric and preperimetric patients.

### Nerve fibre layer thickness

A spectral domain OCT (Spectralis, Heidelberg Engineering, Germany) was used to examine the mean thickness of the RNFL at a circle of 3.4-mm diameter at 768 positions around the ONH [37].

Data of the subjects in the 4 study groups are shown in Table 1.

#### Normal subjects

The study included 19 left eyes of 19 healthy control subjects (9 males, 10 females). They did not show any abnormality in the ophthalmologic evaluation, including slit-lamp inspection, perimetry, tonometry, fundoscopy, and papillometry. The controls underwent the identical diagnostic programme compared with the subjects with open-angle glaucoma and ocular hypertension.

#### Ocular hypertension group

Patients in this group (17 left eyes; 8 males, 9 females) had earlier intraocular pressures above 21 mmHg upon repeated measurements. All of them had a ‘non-perimetric’ visual field result with white-on-white perimetry and normal ONHs.

#### ‘Preperimetric’ glaucoma group

In the ‘preperimetric’ glaucoma group (24 left eyes; 11 males, 13 females), subjects showed glaucomatous abnormalities of the ONH and localized or diffuse loss of the retinal nerve fibre layer (RNFL). Computerized visual field examinations with white-on-white perimetry (Octopus, programme G1) were normal. This ‘preperimetric’ glaucoma group included 21 eyes with primary open-angle glaucoma and 3 eyes with secondary open-angle glaucoma due to pigmentary glaucoma or pseudoexfoliation.

#### ‘Perimetric’ glaucoma group

The ‘perimetric’ glaucoma group (50 left eyes; 29 males, 21 females) included 23 eyes with primary open-angle glaucoma characterized by elevated intraocular pressure higher than 21 mmHg, 15 eyes with secondary open-angle glaucoma with elevated intraocular pressure due to pigmentary glaucoma or pseudoexfoliation, and 12 eyes with normal-pressure glaucoma. All patients of this ‘perimetric’ glaucoma group had glaucomatous ONH damage and local and/or diffuse visual field loss in white-on-white perimetry (mean defect 8.6 ± 5.6).

Patients with ocular hypertension and open-angle glaucoma were treated to reduce the IOP (medicamentous or with surgery, e.g. laser trabeculoplasty).

### Acquisition of video sequences

The video sequences were acquired using a recently developed video ophthalmoscope [32, 33]. The instrument is based on the principle of a fundus camera. An ophthalmic lens produces an aerial image of the retina in the aerial image plane. An objective lens images the aerial image to a light detector with high light sensitivity, either a CCD or CMOS camera. The frame rate was set to 25 frames per second. The aerial image plane contains a field stop to restrict the field of view (20° × 15°) centred to the ONH and a fixation target (small LED or OLED display) to maintain stable fixation of the subject during data acquisition. To achieve high light absorption by blood, the illuminating wavelength was 575 nm (LED). In the pupil plane, the illuminating and imaging light paths are inverted, which simplifies the optical setup [33]. The size of the entire instrument is small (37 cm × 7 cm × 7 cm).

All subjects had a 10-min rest time in the examination room before measurement to reduce the possible influence of physical exertion. After alignment of the instrument to the subject’s eye (with dilated pupil) using a head and chin rest and an adapted XYZ mount of a slit lamp, 3 video sequences were acquired for each subject with a duration of 10 s each. The entire measurement, including alignment of the instrument, took less than 1.5 min. The acquired video sequences were stored for off-line evaluation.

### Calculation of parameters

**Fig. 1:**
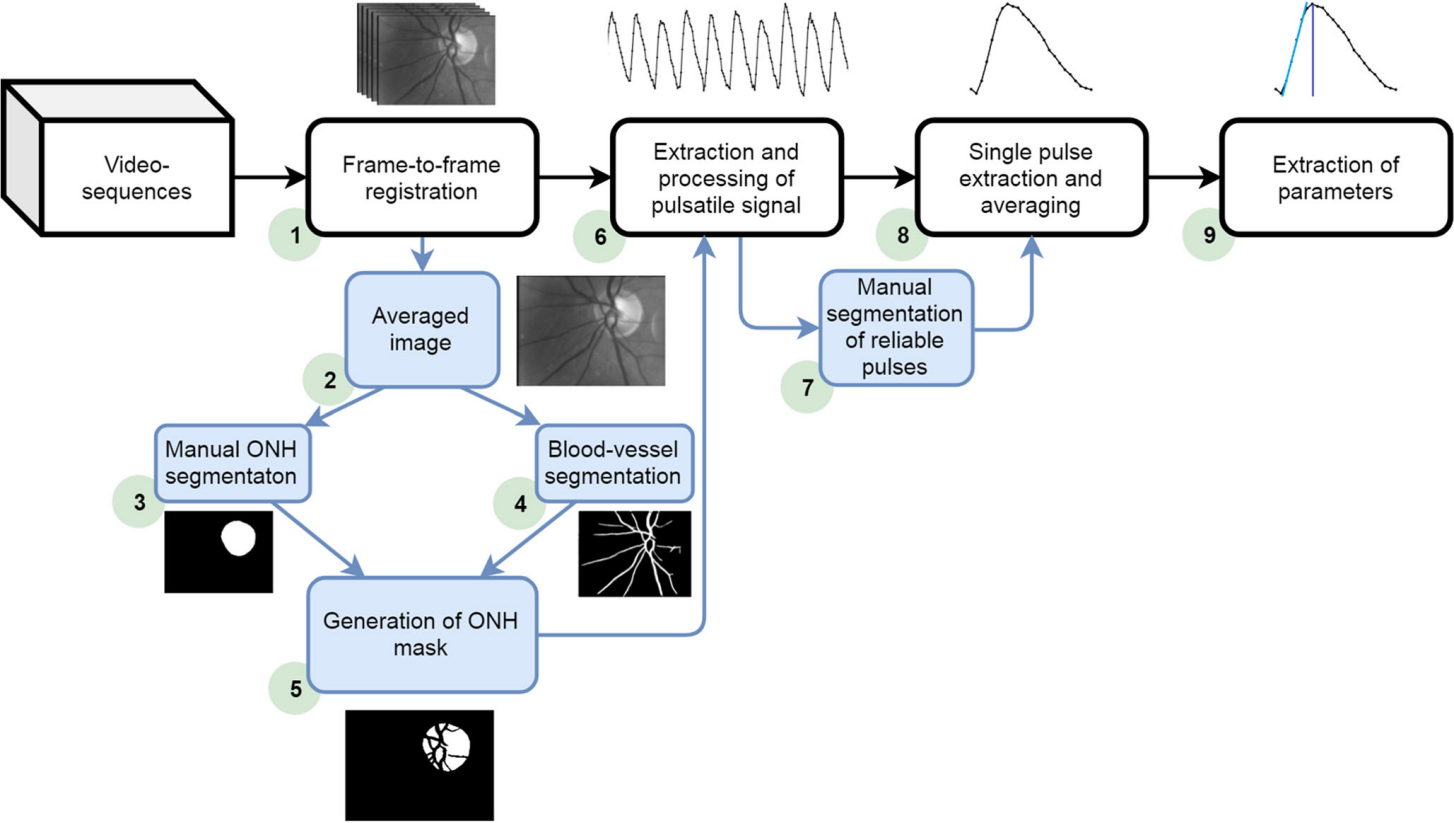
Flowchart to calculate pulse parameters of the plethysmographic signal of the ONH from video sequences. The different steps (1) to (9) are explained in the text

To calculate various blood flow–related parameters (peak amplitude, steepness of ascending part of pulse, time-to-peak, full width at half maximum, and pulse duration, see below), the acquired video sequences were processed offline according to the flowchart shown in Fig. 1. The different steps are described below.

### Suppression of eye movements

The video sequences must be registered to eliminate eye movements during acquisition (step 1). We have developed a method for frame-to-frame registration [38], which effectively suppresses eye shifts and rotation in acquired sequences. This method has been applied to all sequences of subjects included in this study.

The eye movements can also cause specific distortions of some frames and influence the consecutive analysis. These distortions are as follows: motion blur (due to fast eye movement during the exposure time of one frame), out-of-focus blur (due to strong optical aberration, head movement, eye focus changes), and strong eyelid reflections (due to eye blinking).

### Segmentation of structures

All 250 frames of a registered sequence were averaged to create one representative retinal image (step 2), which is used for ONH and blood vessel segmentation. The ONH has been segmented manually to avoid imprecise segmentation in some challenging cases (step 3). Next, the blood vessels have been segmented automatically by a pixel-wise classification-based approach described in [39] (step 4). The segmented blood vessel tree has been further morphologically dilated (with a circular mask 5 pixels in size), which causes a slight increase of the segmented tree to eliminate the close blood vessel surroundings, which might be influenced by blood vessel pulsation and movement. These two binary masks (from ONH segmentation and blood vessel segmentation) are merged together (step 5) to create a new binary mask corresponding only to ONH tissue, further referred to as ONH ROI.

### Calculation of plethysmographic signal

**Fig. 2:**
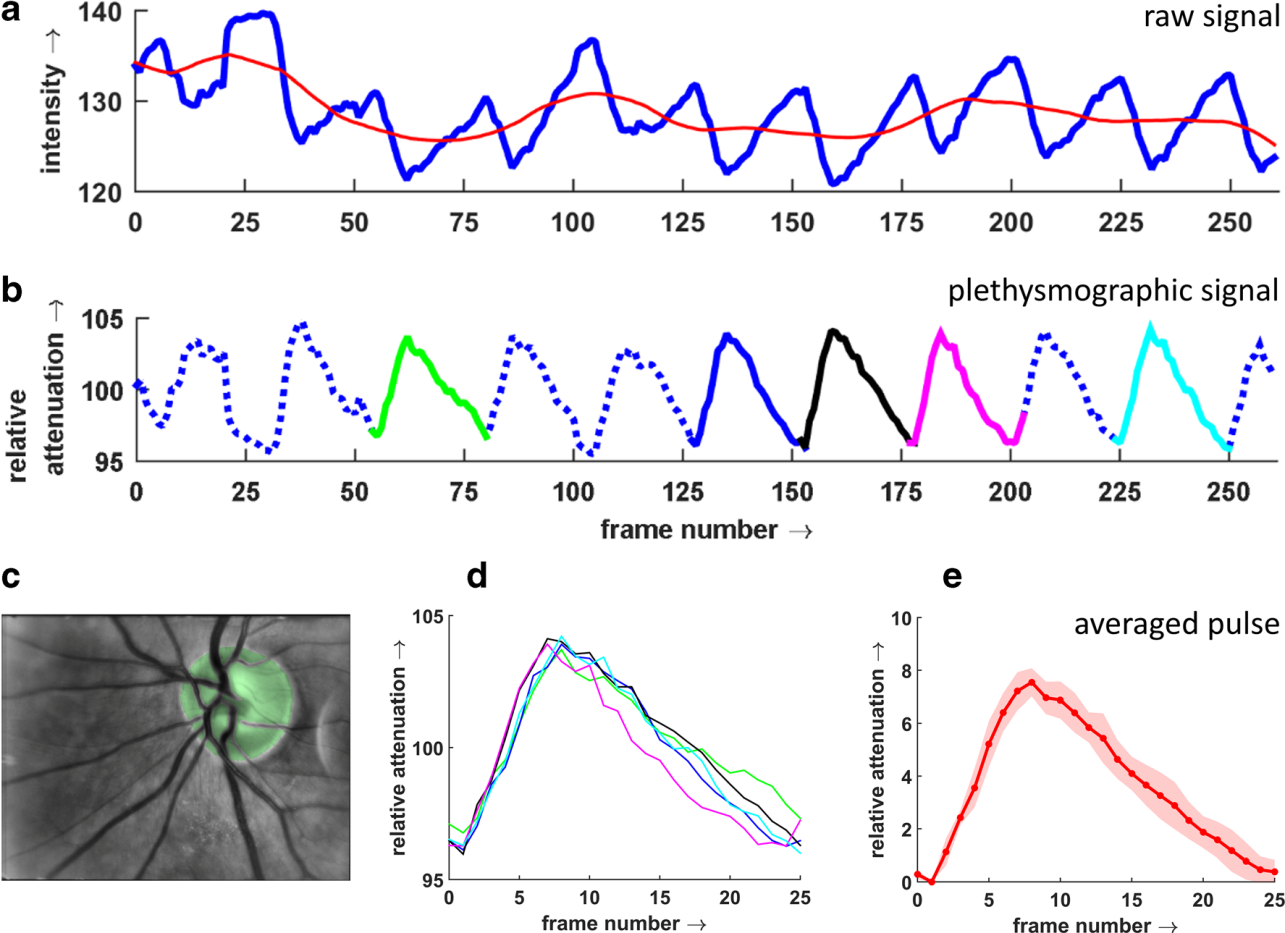
Calculation of averaged pulse from raw (intensity) signal of a normal subject. (A) original pulsatile signal (thick blue line) and calculated trend (red thin line). (B) Calculated plethysmographic signal after trend correction (dotted line) and manually selected pulses (solid parts). (C) Averaged image with segmented ONH ROI (green). (D) Five selected individual single pulses. (E) Averaged pulse was calculated from selected single pulses (red line) and standard deviation (reddish area). Twenty-five frames correspond to 1 s

The binary mask is used to extract the reflected light intensity from ONH ROI (measured as grey levels in acquired frames) (step 6). For each frame of the sequence, the intensity for all pixels within the ONH ROI is averaged. The succession of these values of all frames of a sequence represents the signal of the changing intensity *I*(*n*) (*n*: frame number) due to cardiac cycle–induced light absorption changes (raw signal, Fig. 2A). The raw signal is proportional to the reflected light intensity. The trend-corrected plethysmographic signal *A*(*n*) that is proportional to the absorption due to the changing blood volume is calculated as *A*(*n*) *=* 1 − [*I*(*n*)/*I*_avg_(*n*)], where *I*_avg_(*n*) is the trend signal [32]. The plethysmographic signal shows higher values for higher blood volume (i.e. higher light absorption) and vice versa. To be able to directly display the results in a video sequence, all values are multiplied by 100 [32]. This signal can be interpreted as the relative deviation of light absorption from the average value (value 100) during the cardiac cycle. For instance, the value 105 means an increase of absorption of 5% (Fig. 2B) compared with the average value.

### Selection of pulses

As mentioned above, the eye movements and eye blinking during the acquisition create artefacts that cannot be completely suppressed or removed. Therefore, we decided on manual inspection of each plethysmographic signal and manual selection of reliable pulses (without distortion due to fast eye movement, eye blinks, or light reflection from corneal reflex) for parameter extraction (step 7). The investigator who selected the pulse cycles was masked to the diagnosis. The local minima of undistorted pulses have been labelled and stored for each sequence. Typically, 5 (2 to 7) pulses have been selected (Fig. 2B, D). In the next step, these labels have been used to automatically select these pulses and compute one averaged pulse (step 8). For the averaged pulse, the reference value is the starting point at the beginning of the heartbeat; this means it represents the changing light attenuation during one cardiac cycle. Further, this signal is referred to as the averaged pulse (Fig. 2E).

### Pulse-related parameter extraction

**Fig. 3:**
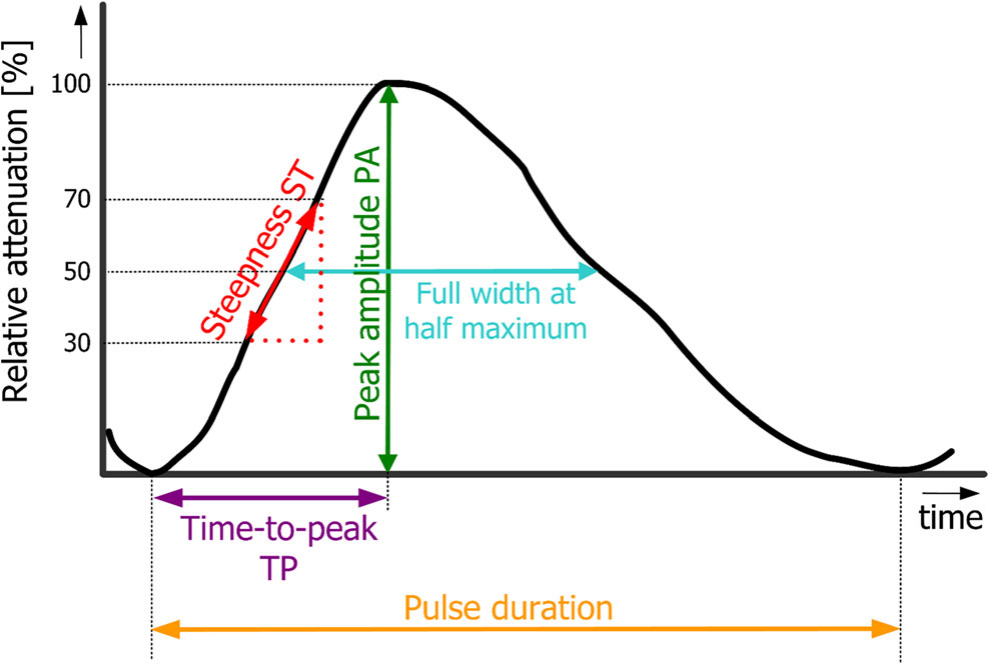
Graphical explanation of calculated parameters from averaged pulse

The following parameters were extracted (step 9) and tested for all averaged pulses (see Fig. 3):

- *Peak amplitude* in %A—the maximum value of the averaged pulse. The unit [%A] is used to show that this signal is related to the absorption. This can be interpreted as the maximum value of blood volume with respect to the beginning of the cardiac pulse.
- *Steepness* of the ascending part in %A/s—calculated as an increase of intensity from 30 to 70% of the value of peak amplitude divided by the corresponding time (as shown in Fig. 3). Other methods of steepness calculation (minimum-maximum value, 47 to 53% value, and maximum value of numerical differentiation) have also been tested and provided similar results. This parameter can be interpreted as the speed of blood volume increase during systole.
- *Time-to-peak* (s) and percentage (%) of pulse duration—time to reach the maximum from the beginning, expressed in seconds and relatively in percentage with respect to pulse duration.
- *Peak width at half maximum* (*FWHM*) in seconds (s) and percentage (%) of pulse duration—width of the peak at 50% of its height, also computed in seconds and relatively in percentage with respect to pulse duration.
- *Pulse duration* in seconds (s)—duration of the averaged pulse.

### Statistics

All statistical analyses were performed using SPSS software (SPSS Inc., version 21, Chicago, IL, USA). The results include means and standard deviations, as well as medians and quartiles, in the boxplot presentation. The normality distribution of the results and equal variances could not be confirmed for all groups. Hence, non-parametric tests were used in the statistical analysis. Comparisons between groups were made using the Mann-Whitney *U* test and the Kruskal-Wallis test. The Spearman correlation analysis was performed to study the association between parameters and mean thickness of RNFL. To take into account the possible error by multiple comparisons, all results were corrected according to Bonferroni. Analysis of variance was used to calculate the coefficient of variation (CV) for two repeated measurements. *p* < 0.05 was considered statistically significant for all tests.

## Results

The reproducibility of the method has been evaluated using repeated measurements for 9 control subjects. The CVs for the pulse parameters amplitude, slope, and duration were 0.086, 0.088, and 0.042, respectively.

**Fig. 4:**
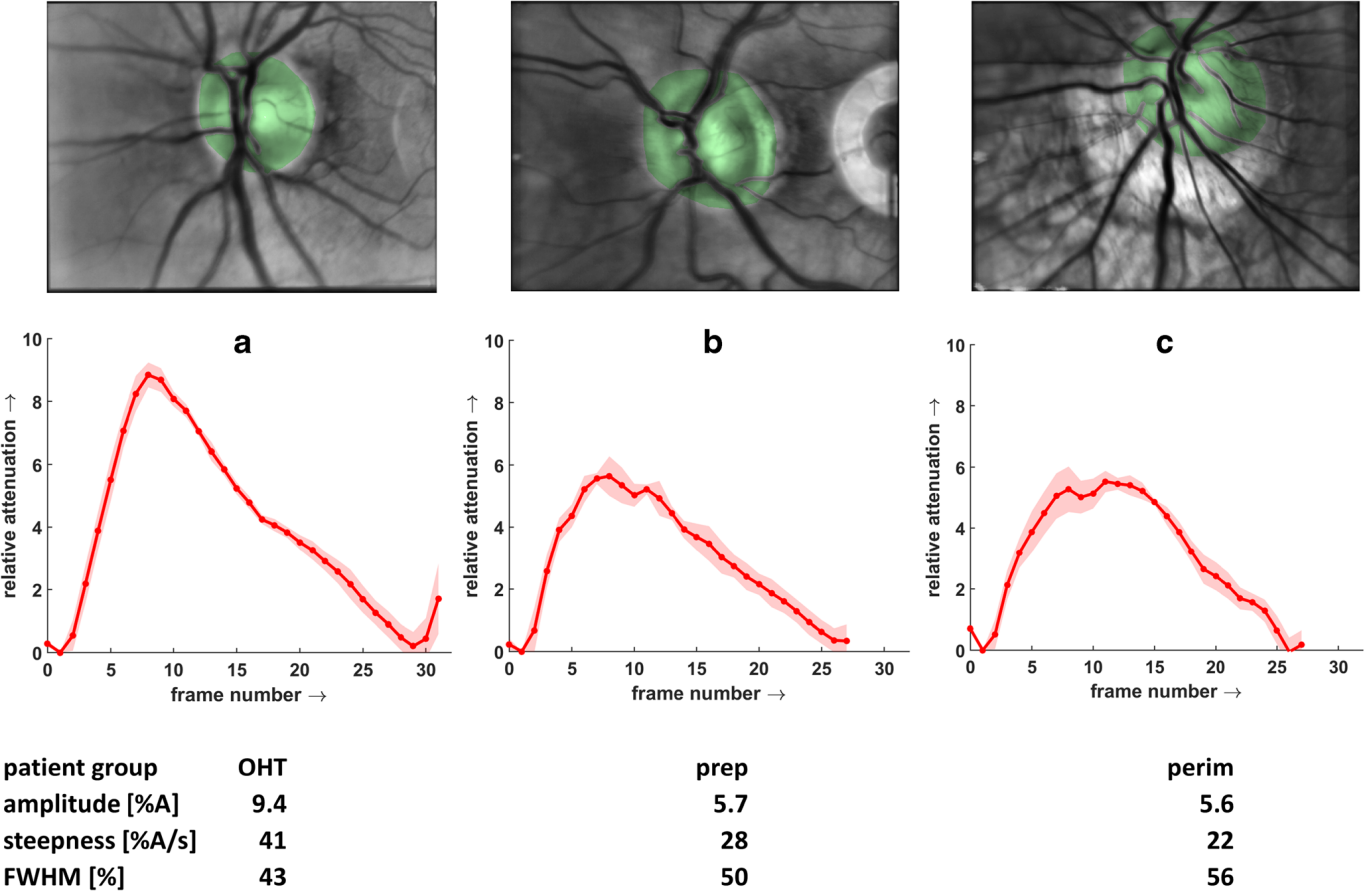
Examples of calculated averaged pulses. Upper row: averaged fundus images of the entire sequence and segmented ONH ROIs (green area). Middle row: averaged pulses (red line) and standard deviations (reddish areas) calculated from the green ROIs shown in the upper row. Lower row: calculated pulse parameters (the bright semicircle on the right side in image B is due to a not correct aligned aperture during this measurement, but it has no influence on the result.) A corresponding example of a normal subject (amplitude 7.6%A, steepness 34%A/s, FWHM 44%) is shown in Fig. 2C and E. Patient groups: OHT, ocular hypertension; prep, preperimetric glaucoma; perim, perimetric glaucoma

Figure 4 shows examples of calculated averaged pulses and corresponding ONH ROI segmentation in averaged images from 3 different patient groups.

Differences in amplitude and waveform, e.g. in the behaviour of the leading and falling edges, can be clearly seen. Figure 4A shows a subject with high peak amplitude (9%A) and a nearly linear rising edge, whereas the subjects in B and C show lower peak amplitude (about 6%A) and deviation from the linearly rising edge.

Table 2 presents the mean values and standard deviations for all pulse parameters in the four subject groups. The table additionally includes the results (*p* values) comparing the groups with the Kruskal-Wallis test. Significant results (*p* < 0.001) were found for steepness and peak amplitude. However, time-to-peak, FWHM, and pulse duration did not differ between groups.

**Table 2:** Mean, standard deviation, and outcome of the Kruskal-Wallis test for parameters in different subject groups. Patient groups: *OHT*, ocular hypertension; *prep*, preperimetric glaucoma; *perim*, perimetric glaucoma; *norm*, normal subjects

Patient group	Number of subjects	Peak amplitude (%)	Steepness (%A/sec)	Time to peak (s)	Time to peak (%)	FWHM (s)	FWHM (%)	Pulse duration (s)
Norm	19	5.86 ± 1.84	29.23 ± 6.72	0.34 ± 0.09	33.09 ± 7.67	0.56 ± 0.12	50.29 ± 8.93	1.05 ± 0.18
OHT	17	6.60 ± 2.41	32.55 ± 9.98	0.30 ± 0.05	32.25 ± 7.30	0.51 ± 0.08	50.68 ± 7.16	0.94 ± 0.16
Prep	24	5.17 ± 1.80	24.27 ± 9.33	0.35 ± 0.08	32.54 ± 5.29	0.55 ± 0.12	47.28 ± 7.72	1.08 ± 0.13
Perim	50	4.06 ± 1.67	18.84 ± 8.26	0.35 ± 0.08	35.88 ± 7.27	0.55 ± 0.09	51.86 ± 7.61	1.00 ± 0.17
Kruskal-Wallis test	*p*	< 0.001	< 0.001	n.s.	n.s.	n.s.	n.s.	n.s.

**Fig. 5:**
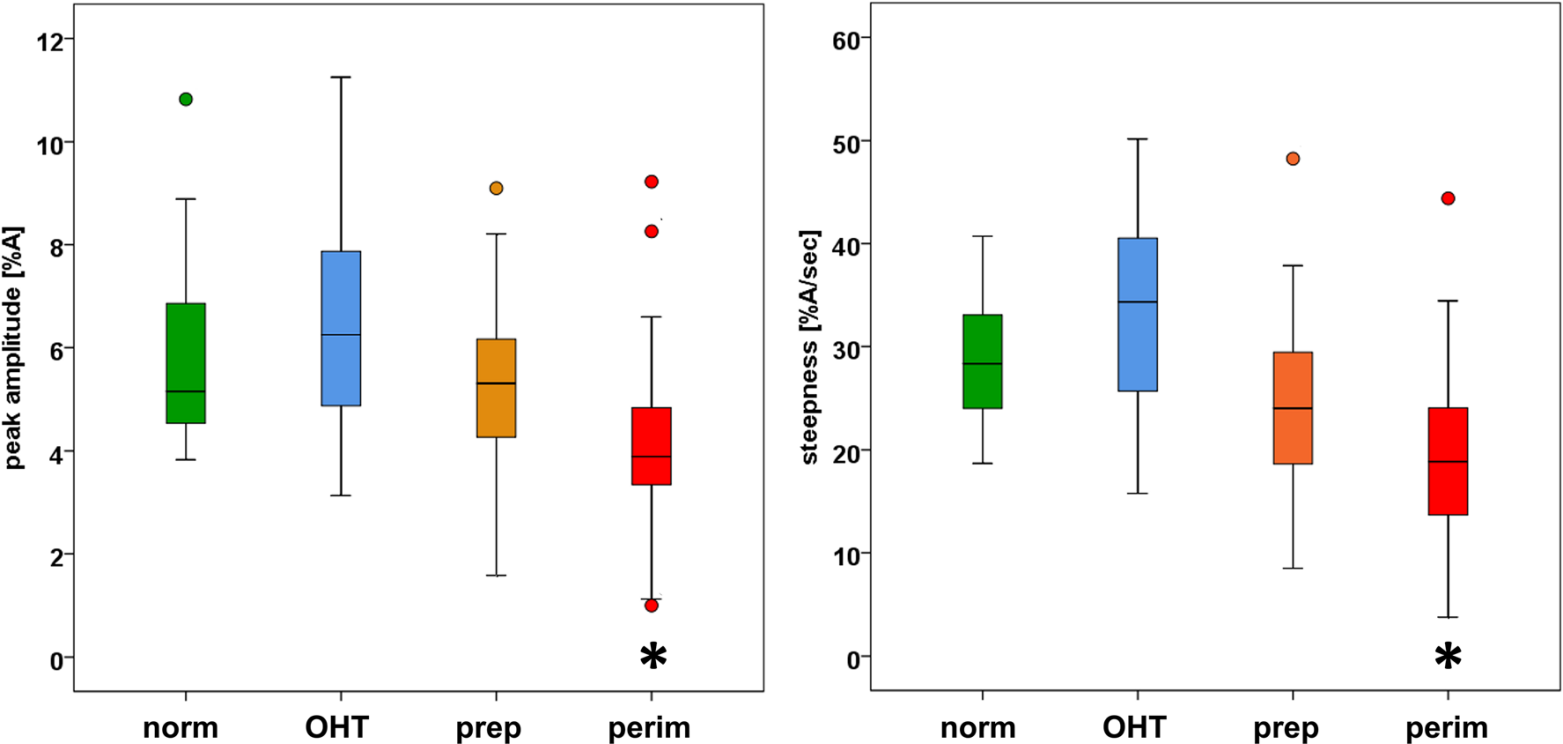
Boxplots showing medians and quartiles of peak amplitude and steepness for the different subject groups. Significant differences (*p* < 0.001) between groups (Mann-Whitney test) in comparison with normal subjects are indicated by asterisks (*). Patient groups: OHT, ocular hypertension; prep, preperimetric glaucoma; perim, perimetric glaucoma; norm, normal subjects

For the parameters with significant differences between groups (peak amplitude and steepness, see Table 2), boxplots including medians and quartiles are presented in Fig. 5. The figures clearly show reductions of peak amplitude and steepness in the more advanced glaucoma groups.

The relationship between the pulse parameters and the severity of glaucoma disease is furthermore shown by correlation analyses for the total patient cohort. The correlation analyses (Spearman test) of mean thickness of RNFL with the pulse parameters revealed significant correlation coefficients (*p* < 0.001) for peak amplitude (*R* = 0.523) and steepness (*R* = 0.568), but not for time-to-peak, FWHM, and pulse duration (not shown graphically). Separate correlation analyses in males and females showed similar relationships between slope and RNFL thickness for both groups (male *R* = 0.31, female *R* = 0.29).

**Fig. 6:**
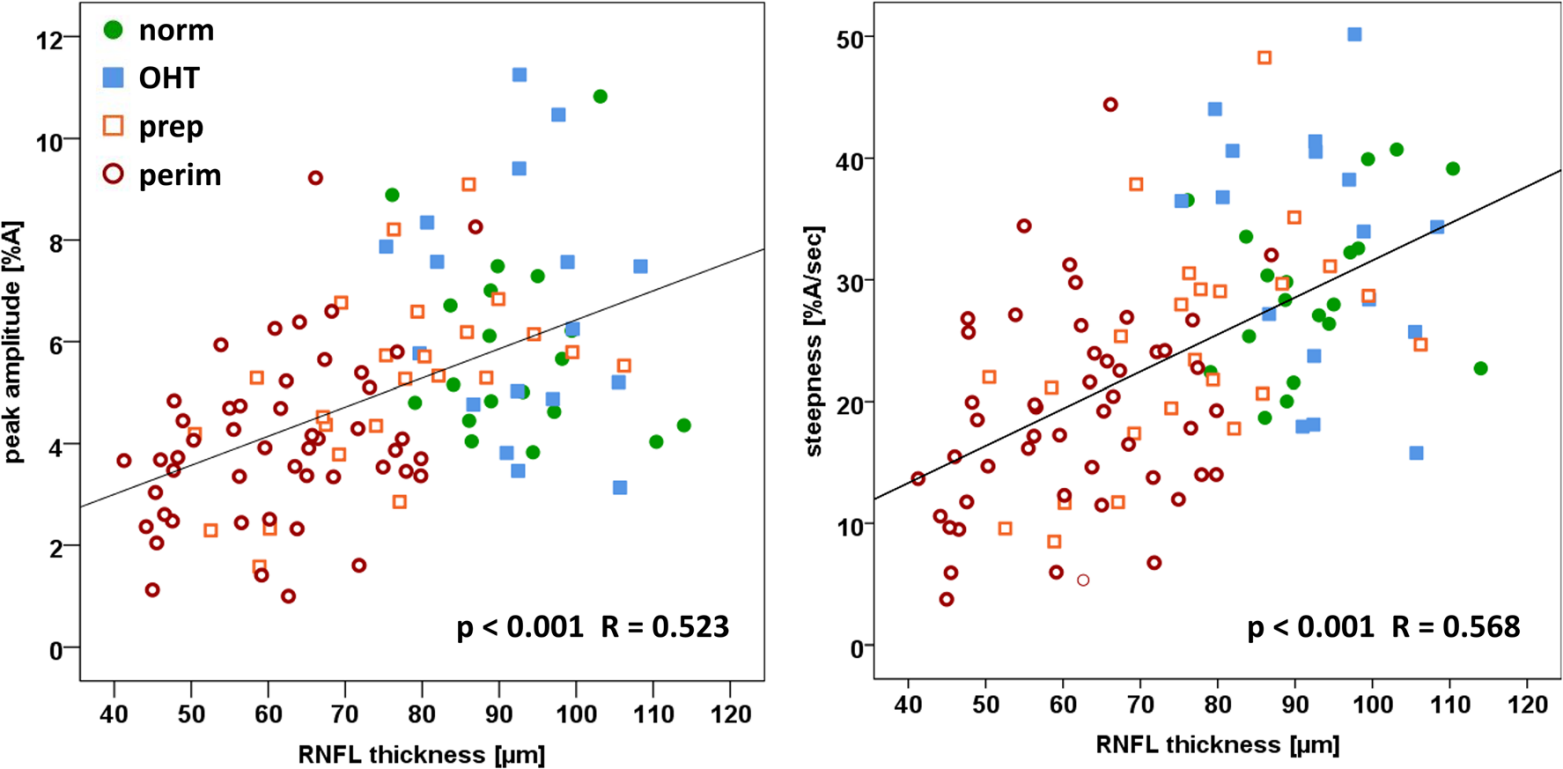
Scatterplots of peak amplitude and steepness as functions of RNFL mean thickness, including regression lines. The Spearman test indicates a significant association between peak amplitude and steepness with RNFL thickness. Patient groups: OHT, ocular hypertension; prep, preperimetric glaucoma; perim, perimetric glaucoma; norm, normal subjects

The scatterplots showing peak amplitude and steepness as functions of RNFL mean thickness are presented in Fig. 6. Regression lines indicate reductions of peak amplitude by 0.6%A and steepness by 3%A/sec for every 10-μm loss of RNFL thickness.

## Discussion

The method described in this paper measures the pulsatile component of the changing blood volume in the capillaries of the ONH tissue. Our results show reductions of peak amplitude and steepness with increasing severity of glaucoma and a high correlation of these two parameters with RNFL thickness. The peak amplitude can be related to the amount of pulsatile blood volume flowing through examined tissue and depends on both tissue capillary density and the amount of blood flowing through these capillaries. The steepness of our pulsatile component can be interpreted as the velocity of blood filling, i.e. how fast the tissue is filled by blood during systole. Our findings show a reduction of the values of these two parameters in glaucoma subjects. Although our pulse parameters are based on light absorption related to blood volume changes and not on particle movement, we showed that our first results of ONH perfusion assessment are in agreement with the results obtained by other, more sophisticated modalities such as OCT-A and LSF:

Using OCT-A, Jia et al. 2012 [16] showed large flow index and vessel density reduction for three preperimetric glaucoma subjects, particularly in temporal regions of the ONH. Significant ONH perfusion reduction has also been found by Chen et al. [21] in glaucomatous eyes using optical microangiography (OMAG)–derived ONH perfusion metrics (blood flux, vessel density area, normalized flux) from prelaminar tissues (tissues between the internal limiting membrane and the anterior surface of the laminar cribrosa). Significant negative correlations between ONH perfusion and glaucoma severity have also been found to be statistically significant. Wang et al. [22] explored ONH perfusion in open-angle glaucoma (OAG) subjects and found a statistically significant correlation between RNFL thickness and ONH flow index in OAG subjects (*n* = 62), but not in healthy subjects (*n* = 20). The mean RNFL thicknesses in mild, moderate, and severe glaucoma groups were 86.5 μm, 77.0 μm, and 64.0 μm, respectively, corresponding to the RNFL thickness in our study. Lévêque et al. [40] also found this statistically significant correlation (RNFL thickness vs. blood vessel density in both, whole and temporal ONH part) for advanced glaucoma cases included in their study (mean RNFL value 66.48 ± 12.6 μm). A comprehensive review of OCT-A in glaucoma is given in [41], where authors reviewed various studies suggesting that OCT-A-derived perfusion parameters decrease in ONH, peripapillary area, and macular area.

Laser speckle flowgraphy is another modality used to assess ONH perfusion. Although a recent comparison [42] of OCT-A and LSF showed that vessel density (from OCT-A) has a higher glaucoma diagnostic ability compared with LSF parameters in patients with normal tension glaucoma (NTG), the advantage of LSF is that it has an additionally high temporal resolution. This enables analysis of speckle-induced pulsatile changes, i.e. shape of the waveforms from different retinal regions, on a pulse-by-pulse level. Recent LSF-based studies describe changes of these waveforms (and related parameters) for glaucoma subjects. Shiga et al. [31] described a delay in the peak of the MBR waveform, a flattened waveform, and reduced MBR amplitude for normal tension glaucoma (NTG) subjects in the ONH capillary area only. Mursch-Edlmayr et al. [28] found a reduced blood flow in the vessels supplying the retina assessed by MBR from ONH in NTG subjects. They also evaluated the shape of the MBR curve using several specific parameters and found a flatter waveform for NTG subjects, which means a lower MBR_avg_ and a lower flow acceleration index. The results of these studies are similar to our findings—lower waveform amplitude and lower steepness in glaucoma groups. Although the results from different studies are not coherent, it is evident that the waveform analysis of blood-induced pulsatile patterns extracted from ONH capillaries and obtained by different principles can contribute to an improved understanding of glaucoma vascular dysfunction. Furthermore, there are studies (e.g. [29]) showing that MBR from the ONH capillaries is significantly correlated with the RNFL thickness in the superior, inferior, and temporal ONH quadrants.

The waveform of the pulsatile signal contains important information. It can be affected by different kinds of provocation, such as increased intraocular pressure [43] and flicker light stimulation [44, 45], and also by diseases, such as carotid artery stenosis (CAS) [46–50] and glaucoma [31, 51]. Some of the above-mentioned results are based on the measurement of the pulsatile signal of the intraocular pressure and not on the blood volume absorption signal. However, it was shown for the capillaries of the finger that the blood volume pulse and the radial pressure pulse can be represented by a single mathematical transfer function. This implies that the information in the blood volume pulse is similar to that contained in the radial pressure pulse [52], so that it has to be expected that changes in the IOP pulse can also be seen in the blood volume pulse.

OBF can be affected by IOP, blood pressure, heart rate, systemic diseases, cardiovascular diseases, medication (especially antihypertensive and hypotensive medication, topical medication), haemoglobin concentration, haematocrit, and vessel stiffness. However, the fact that most of the above-mentioned parameters are naturally identical on both eye sides allows the detection of differences that are caused by asymmetric IOP and/or asymmetric stenosis of carotid arteries (CAS). As diseases such as glaucoma [53–55] and CAS often start asymmetrically [50], the exact comparison of the parameters of the plethysmographic signals between eye sides could reveal deviation from normal behaviour caused by beginning CAS and/or increased IOP as a sign of beginning glaucoma. Using a binocular video ophthalmoscope (consisting of two identical video ophthalmoscopes as described here), it is possible to measure both eyes simultaneously [32]. Providing an exact synchronization of the cameras of the two instruments, parameters of the plethysmographic signal that are caused by the same heartbeat can be exactly compared. In this way, even a small time shift between sides can be measured. The first results show that, for instance, the plaque of the *carotis communis* can cause a small time delay between eye sides [32]. Therefore, it could be possible to detect pathologic differences between the signals on both eye sides without knowledge of the other OBF-influencing parameters.

Nevertheless, a possible limitation of our study is that many parameters that can influence OBF are not considered. However, at the present state of our development, there are too few subjects to draw reliable clinical results on the influence of these parameters.

Future work will involve measuring the influence of the above-mentioned OBF-related parameters on the plethysmography signal in a larger subject group and also the comparison of the plethysmographic signals of right and left eyes to detect deviation from symmetry as an early sign of the initiation of diseases such as glaucoma or carotid artery stenosis.

## Conclusion

The presented low-cost video-ophthalmoscope allows the measurement of the plethysmographic signal of the ONH tissue and the calculation of different perfusion-related parameters. Our findings show that the values of peak amplitude and steepness are significantly reduced in patients with perimetric glaucoma in comparison with normal subjects, and both parameters showed significant correlations with RNFL thickness. This is in line with the results of other clinical techniques such as OCT angiography and laser speckle flowmetry, which also showed reduced blood flow in ONH tissue in glaucoma. A possible limitation of our study is that the influences of OBF-related parameters such as blood pressure and heart rate on these measurements are not considered. The influence of the latter parameters might be lower if simultaneous measurements of the right and left eyes would be compared to detect deviations from symmetry. Due to the small size of the instrument, it is possible to use two video-ophthalmoscopes to measure both eyes simultaneously. This binocular setting will allow comparisons of the plethysmographic signals of both eye sides induced by the same heartbeat and quantification of the asymmetry of ONH blood flow among both eyes with high spatial and temporal resolution.

## Data Availability

Not applicable
